# Warm Air Delivery in Adhesive Application: Effect on Bonding Performance and Morphological Outcomes

**DOI:** 10.3390/biomimetics9040194

**Published:** 2024-03-24

**Authors:** Rim Bourgi, Naji Kharouf, Carlos Enrique Cuevas-Suárez, Monika Lukomska-Szymanska, Khalil Kharma, Fabienne Hajj Moussa, Manar Metlej, Youssef Haikel, Louis Hardan

**Affiliations:** 1 Department of Restorative Dentistry, School of Dentistry, Saint-Joseph University, Beirut 1107 2180, Lebanon; rim.bourgi@net.usj.edu.lb (R.B.); khalil.kharma@usj.edu.lb (K.K.); 2 Department of Biomaterials and Bioengineering, INSERM UMR_S 1121, University of Strasbourg, 67000 Strasbourg, France; dentistenajikharouf@gmail.com (N.K.); youssef.haikel@unistra.fr (Y.H.); 3 Department of Endodontics and Conservative Dentistry, Faculty of Dental Medicine, University of Strasbourg, 67000 Strasbourg, France; 4 Dental Materials Laboratory, Academic Area of Dentistry, Autonomous University of Hidalgo State, San Agustín Tlaxiaca 42160, Mexico; 5 Department of General Dentistry, Medical University of Lodz, 92-213 Lodz, Poland; monika.lukomska-szymanska@umed.lodz.pl; 6 Laboratoire de Pharmacologie, Pharmacie Clinique et Contrôle de Qualité des Médicaments, Faculty of Pharmacy, Saint-Joseph University, Beirut 1107 2180, Lebanon; fabienne.hajjmoussa@usj.edu.lb; 7 Department of Prosthetic Dentistry, School of Dentistry, Saint-Joseph University, Beirut 1107 2180, Lebanon; manar.metlej@net.usj.edu.lb; 8 Pôle de Médecine et Chirurgie Bucco-Dentaire, Hôpital Civil, Hôpitaux Universitaire de Strasbourg, 67000 Strasbourg, France

**Keywords:** adhesive systems, dentin bonding, direct restorations, micro-tensile bond strength, solvent evaporation

## Abstract

Solvent evaporation within an adhesive layer is a crucial step during a bonding process. The aim of this current research was to test whether the use of different air temperatures (20 °C, 40 °C, and 60 °C) for solvent evaporation improves the performance of four adhesive systems to dentin. Sixty non-carious human molar teeth were randomly prepared for micro-tensile bond strength (μTBS) tests. Four different adhesive systems, Prime&Bond Universal (PBU), OptiBond Universal (OBU), OptiBond FL (OBFL), and Clearfil SE (CSE), were applied following the manufacturer’s instructions. Three groups based on the air-drying temperature were used: solvent evaporation was performed with either of warm (40 °C), (60 °C), and cold air as control group (20 °C) for 10 s at a distance of 5 cm. In all bonded surfaces, three resin composite (Reflectys, Itena Clinical, Paris, France) layers of 2 mm thickness were built up. The resin–dentin samples were kept in distilled water at 37 °C for 24 h and 6 months, respectively, before μTBS testing. Failure analysis, scanning electron microscopy of resin–dentin bonded interface, and solvent evaporation rate were tested as secondary variables. All analyses were conducted using a significance level of α = 0.05. Bond strength (BS) values were similar among all the adhesive systems used (*p* > 0.05). Also, the aging factor did not affect the BS (*p* > 0.05). Only the factor of temperature used for solvent evaporation resulted in a statistically significant effect (*p* < 0.05), with the temperature of 60 °C being the highest value (*p* < 0.05). A failure mode evaluation revealed mostly adhesive or mixed modes of failures in all the different temperatures of air used for the solvent evaporation of each adhesive system. The thickness of the adhesive layer and the creation of resin tags varied amongst the temperatures evaluated. For all adhesive systems tested, the use of 40 °C or 60 °C air for solvent evaporation led to an increased mass loss. Warmer temperatures for solvent evaporation contributed positively to bonding performance, enhancing both the quality of the adhesive layer and its interaction with the dentin tissue. Optimizing solvent evaporation with warmer air temperatures (40 °C and 60 °C) significantly improved µTBS, offering a practical means to enhance the quality and longevity of adhesive restorations in esthetic dentistry.

## 1. Introduction

The backstory of adhesive systems has resulted in a significant improvement in the bonding procedure, allowing for simultaneous demineralization and resin penetration into the hard-dental tissues [1]. Presently, contemporary dental adhesives are primarily categorized into two main groups, providing clinicians with the option to choose between etch-and-rinse (ER) or self-etch (SE) adhesives [2]. The acidic monomers in the SE dental adhesives demineralize dentin in the same way that an etchant/conditioner used in the ER dental adhesives does [3]. As perhaps the most recent generation of bonding agents, universal adhesives (UAs) have been developed with the goal of minimizing operational difficulties. In addition, these systems optimize bond strength (BS) and are capable of connecting to a variety of substances [4].

The term “adhesive system” refers to a material that contains the key components agents (etchant, primer, and bond) in different combinations [5]. Irrespective of their classification, dental adhesives consist of hydrophobic and hydrophilic methacrylate monomers, along with photo-initiators, co-initiator systems, and volatile solvents. Co-polymers including polyacrylic acid, fillers, and silane molecules are also used in the most modern UAs. Solvents, being a crucial component of adhesive systems, play a vital role in achieving optimal bonding to dental substrates [2]. Ethanol, acetone, and water are the frequently used solvents inside adhesives, and up to 80% of solvents in weight were included inside the composition of commercial dental adhesives [6]. Some adhesive systems (particularly SE adhesives) contain more than one solvent (for example, ethanol and water). Tert-butanol is a solvent that has been lately incorporated into commercial dental adhesives. While previous studies have shown promising results with alternative solvents like tetrahydrofuran and dimethyl sulfoxide [7,8], they have not yet been utilized in commercial resin adhesives.

Solvents serve as a route for transporting co-monomer blends and initiators to the dental substrate. They dilute the monomers with a relative viscosity, aiding in their penetration and spreading into the dentin structure [9]. Consequently, an adequate ratio of solvents and monomer must be considered necessary for obtaining a high dentinal BS. Any extra solvent within the bonded interface should be evaporated by air-drying [10]. Otherwise, several detrimental consequences could be seen, such as the obstruction of the polymerization of resin monomers, facilitating the degradation of resin–dentin-bonded interfaces, as well as preventing the penetration of resin into the demineralized dentin [11]. All in all, voids within the interface of an adhesive are created, thus decreasing the BS [12]. Increasing solvent content not only dilutes the co-monomer mix but also reduces the concentration of the photo-initiator. This leads to a decreased conversion of resin monomers [11].

The evaporation of solvents from resin blends can be attained either by permitting an evaporation period between adhesive application and photopolymerization or by air-drying by means of a triple syringe. By doing so, several factors influence solvent evaporation, including the type of solvent and co-monomer, operator skill, syringe-to-tooth distance, and air temperature [9,11]. Various methods have been proposed to improve solvent evaporation from dental adhesives, including prolonging adhesive application time, applying multiple adhesive layers, extending adhesive polymerization, and employing active adhesive application techniques. Additionally, some efforts have made in using a warm air stream to facilitate solvent evaporation [13].

High air temperatures might activate molecules for better monomer and adhesive polymerization, which might reduce the dentin collagen matrix degradation [9]. Previously, it was demonstrated that a longer time of warm air evaporation with 38 °C helps in solvent evaporation with an increase in dentin BS [14]. Particularly, when using an air temperature of 60 °C, an increase of around 20% in the resin–dentin bonds could be detected [12]. This could be attributed to the heat from the warm air, which raises the kinetic energy of solvent molecules, facilitating the vaporization of the residual solvent and water [13]. Prior investigations have explored warm air temperatures ranging from 37 °C to 80 °C, indicating the need for determining the ideal temperature for warm air blowing [9,10,12,13]. Warm air temperatures between 40 °C and 60 °C were found to be effective for enhancing solvent evaporation. Nonetheless, the 60 °C temperature was better for stable BS and minimized bond breakdown [13].

To the best of the authors’ knowledge, specific devices for delivering warm air for dental use are scarce, so a prototype of therapeutically relevant warm air blowing was built and tested in this work. The aim of this current research was to test whether the use of different air temperatures (20 °C, 40 °C, and 60 °C) for solvent evaporation improves the performance of four adhesive systems to dentin. For this purpose, a prototype device for warm air delivery was developed. Based on the null hypothesis, variations in air temperatures for solvent evaporation were presumed to have no impact on the BS or morphological properties of various adhesive systems to dentin.

## 2. Materials and Methods

### 2.1. Tooth Selection

After receiving approval from the ethical committee of the dental faculty at the Saint-Joseph University of Beirut, Lebanon (FMD-221; file number: #USJ-2022-140), this study was conducted in accordance with the guidelines outlined in the approved protocol.

In addition, the simple randomization procedure utilized a computer-generated random sequence from www.randomizer.org (1 March 2023) to ensure an unbiased and equitable allocation of teeth, thus improving internal validity and mitigating selection bias.

In this respect, sixty (n = 60) non-carious human molar teeth were collected and randomly used. All teeth were cleaned by means of an ultrasonic scaler and polished in a rubber cup attached to a low-speed motor (MotorTurbo & E-ASP1, Eighteeth, Changzhou, China). Then, the teeth were stored in a solution of 0.2% sodium azide at 4 °C after they had been cleaned and polished [15]. The teeth selected for the current study were recently extracted for orthodontic or periodontal reasons and were free of caries, abrasion facets, cracks, damage due to extraction, and fluorosis.

### 2.2. Dentin Specimen Preparation

After mounting the teeth in gypsum blocks, the occlusal third of the crowns was removed using an orthodontic grinder (Essencedental, Araraquara, SP, Brazil). Subsequently, a consistent smear layer was created on all mid-coronal dentin specimens by sanding with a P320 silicon carbide sandpaper (SiC) for 1 min under running water. The surfaces were then rinsed with distilled water for 30 s before the application of the adhesive and resin composite. Plus, by using a stereomicroscope (Stereo-zoom S8, Leica, Heidelberg, Germany), the exposed dentinal surfaces were assessed for the absence of enamel.

### 2.3. Bonding Procedures

Three groups based on the air-drying temperature were used: solvent evaporation was achieved with either of warm (40 °C), (60 °C), and cold air as control group (20 °C) for 10 s at a distance of 5 cm. A digital thermostat was used to verify the temperature degree and its consistency throughout the blowing time. In all three cases, the air stream was generated by means of a device (gun) for delivering warm or cold air. The air stream had a speed of 5.50 m/s, with an air flow rate of 0.0138 m^3^/s. The device is a prototype that was manufactured and tested according to ISO standards. This prototype employs pressure to propel dry air through a tube. The swirling system’s pressure causes air molecules to agitate, generating heat that warms the air gently as it flows out from the tip orifice.

**Figure 1:**
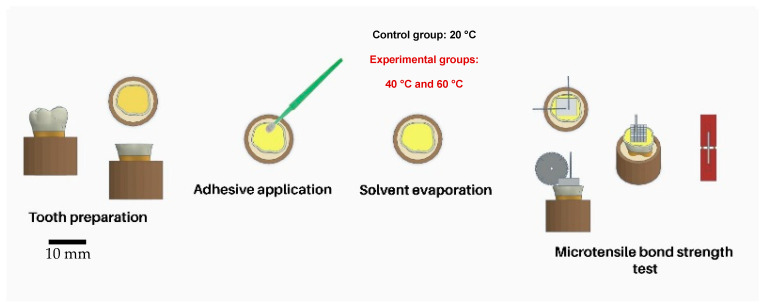
Illustration of the three groups tested in the current study based on the air-drying temperature.

The number of teeth per group (n = 5/air temperature) was estimated based on a previous study that evaluated the effect of warm air application for solvent evaporation on the BS to dentin in a comparative study design [16], an 8.8 minimum detectable difference in means, a 2.9 standard deviation (SD), a power of 0.8, and a = 0.05. The specimen preparation protocol is schematically described in Figure 1.

Four different adhesive systems based on different solvents were used in this research as follows: two UAs (water–isopropanol-based adhesive, the Prime&Bond Universal, PBU, Dentsply DeTrey GmbH, Konstanz, Germany; acetone–ethanol-based adhesive, the OptiBond Universal, OBU, Kerr Co, Orange, CA, USA); one three-step ER adhesive system based on water–ethanol, the OptiBond FL (OBFL, Kerr Co, Orange, CA, USA); and one two-step SE adhesive system based on water, the Clearfil SE (CSE, Kuraray Noritake Dental Inc., Tokyo, Japan). Blinding was not applicable. An overview of the chemical compositions of the adhesive systems examined in the present study is presented in Table 1.

After applying one coat of each adhesive system (Table 1) for 20 s using a microbrush applicator (Kerr, Orange, CA, USA), the air-blowing (based on different air-drying temperature) of the adhesive layers was photopolymerized for 20 s at room temperature (1000 mW/cm^2^, CuringPen-E (Eighteeth, Changzhou, China)). Moreover, a single operator (RB) who completed the adhesion method standardized the level of force to be delivered during the rubbing action. Each bonded surface was constructed in three layers using Reflectys resin composites (Itena Clinical, Paris, France), with each layer being 2 mm thick. Photopolymerization was performed for 20 s per layer at a vertical distance of 1 mm from the dentinal substrate using the same light-curing unit. The resin–dentin samples were kept in distilled water at 37 °C for 24 h and 6 months, respectively, before μTBS testing [17].

### 2.4. Micro-Tensile Bond Strength Testing

After various distilled water storage periods (24 h and 6 months), the restored teeth were sectioned occluso-gingivally using a low-speed precision cutting machine (EXAKT Vertriebs GmbH, Norderstedt, Germany) to produce resin–dentin slabs with a cross-sectional area of approximately 1.0 mm^2^. The μTBS was performed in agreement with ISO/TS 11405. The upper half of the slab consisted of the resin composite, while the lower half was formed by the underlying dentinal part.

Each tooth yielded approximately twelve slabs, which were kept moist until testing. These slabs were then randomly divided into two subgroups, with μTBS testing conducted after 24 h (six slabs/tooth = thirty slabs for five teeth) and 6 months (six slabs/tooth = thirty slabs for five teeth) in each subgroup. The results obtained from the six slabs tested for each tooth were averaged, and the resulting mean was used for statistical analysis [17]. Specimens stored for 6 months were kept in distilled water at 37 °C with weekly replacement. Each slab was stuck to a Geraldeli’s jig by means of a glue (Zapit, Dental Ventures of North America, Corona, CA, USA) and exposed to a tensile load test using a universal testing machine (YLE GmbH Waldstraße, Bad König, Germany) with a crosshead speed of 1 mm/min and a load cell of 500 N. The measurements of each failed specimen were recorded using a digital caliper (Model CD-6BS Mitutoyo, Tokyo, Japan) with 0.01 mm of exactness. Accordingly, the μTBS was determined by dividing the fracture force in newtons (N) by the bonded area in square millimeters (mm^2^) and expressed as megapascals (MPa).

### 2.5. Failure Analysis

**Figure 2:**
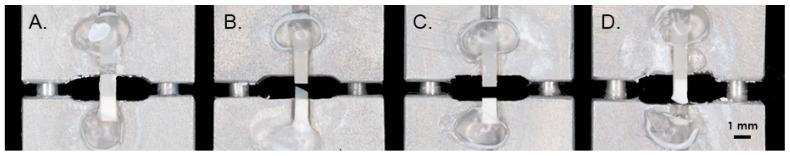
Representative images of the failure mode after the bond strength test. (**A**) Cohesive in resin, (**B**) mixed, (**C**) adhesive, and (**D**) cohesive in dentin.

Each fractured portion’s failure mode was fixed to aluminum stubs and examined using an optical numeric microscope (Keyence, Osaka, Japan) with a magnification of 150×. To calculate the percentage of each fractured area and to designate the type of failure, VHX-5000 software was used. If the bonded interface failed between the resin composite and dentin, the failure was classified as adhesive; if the bonded interface failed in the resin composite or dentin, the failure was classified as cohesive; and if a combination of both adhesive and cohesive failures was recorded, the failure was considered as mixed (Figure 2).

### 2.6. Scanning Electron Microscopy of Resin–Dentin-Bonded Interface

The ultrastructural observation of the restorative–dentin interface (three slabs from each subgroup were kept in hermetic boxes and stored in dry states) was accomplished using scanning electron microscopy (SEM). Specimens were prepared as previously stated in the above test session. Following that, the sample surface underwent polishing using SiC abrasive discs with grit sizes of 1200, 2400, and 4000. Subsequently, 37% phosphoric acid was applied to the polished surfaces for 10 s, followed by a 10 s rinse with distilled water and drying. The samples were then immersed in a 2.5% sodium hypochlorite solution for 3 min to address the smear layer and observe dentinal infiltrations. After rinsing, the specimens underwent dehydration using a graded series of ethanol solutions (25%, 50%, 75%, and 100%). They were then sputter-coated with a gold–palladium alloy (20/80 weight % ratio) for 120 s using a sputtering device (Hummer JR, Technics, CA, USA). Finally, the samples were analyzed with a Quanta 250 FEG SEM (FEI Company, Eindhoven, The Netherlands, 10 kV) at a magnification of 1500×, with a working length of 10 mm to observe the resin–dentin bonded interface.

### 2.7. Solvent Evaporation Rate

Around 10 µL of each adhesive system, equivalent to about one coat using a saturated microbrush, was aspirated with a micropipette (Pipetman, Gilson, NY, USA) from the original flat container and transferred to small light-proof glass containers of known weight. Subsequently, they were promptly placed on an analytical balance (Mettler, type H6; Columbus, OH, USA; capacity up to 160 g), and the baseline mass was recorded to the nearest 0.0001 mg. Different air-drying temperatures (20 °C, 40 °C, and 60 °C) were then applied with the device for every 10 s before placing the adhesive into the analytical balance, at a distance of 5 cm from the container, and the residual mass of all the adhesive systems tested was recorded until the container stopped losing weight to measure the maximal amount of evaporable mixtures. To eliminate the influence of light radiation, an electronic balance was covered with suitable light filters. Five samples (n = 5) of each adhesive under each air-drying condition were analyzed. The percentage of mass loss, relative to the mean baseline recording, was calculated for each tested specimen.

### 2.8. Statistical Analysis

Data from BS were analyzed using the Sigma Plot (Version 14, Systat, San Jose, CA, USA) software. Previously, the data were subjected to analysis to assess the normal distribution and homogeneity of variance. To evaluate the influence of the adhesive system and the temperature used for solvent evaporation on the µTBS to dentin, a two-way analysis of variance (ANOVA) was conducted. The BS was evaluated after 24 h and 6 months of storage, with multiple comparisons conducted using Tukey’s post hoc test. A significance level of α = 0.05 was employed for all analyses.

## 3. Results

### 3.1. Micro-Tensile Bond Strength Testing

Table 2 shows the values obtained for the µTBS after 24 h of aging according to the adhesive and the temperature used for solvent evaporation. The results show that the adhesive factor was not statistically significant (*p* = 0.052), while the temperature had a statistically significant effect (*p* < 0.001). The interaction between the factors was not statistically significant (*p* = 0.880). The temperature for solvent evaporation was only statistically significant for the adhesive PBU, where the differences between 20 °C and 60 °C were significant (*p* = 0.025). The rest of the comparisons were not statistically significant (*p* > 0.05).

Table 3 shows the values achieved for the µTBS after 6 months of aging regarding the adhesive and the temperature used for solvent evaporation. The statistical analysis revealed that only the factor of temperature for solvent evaporation was statistically significant (*p* < 0.001), while the adhesive factor (*p* = 0.369) and the interaction between the factors (*p* = 0.821) were not statistically significant. The post hoc analyses showed that, only for the CSE adhesive, there were statistically significant differences between the 20 °C and 60 °C air temperatures (*p* = 0.008). The rest of the comparison were not statistically significant (*p* > 0.05).

The mean and SD for the µTBS of all the adhesives tested according to the different temperatures of air used for solvent evaporation and storing time are presented in Table 4.

For all air temperatures, there were no statistically significant differences between the 24 h and 6 months of aging (PBU: *p* > 0.05; OptiBond Universal (OBU): *p* ≥ 0.218; CSE: *p* ≤ 0.090; OptiBond FL (OBFL): *p* < 0.139).

### 3.2. Failure Mode Analysis

**Figure 3:**
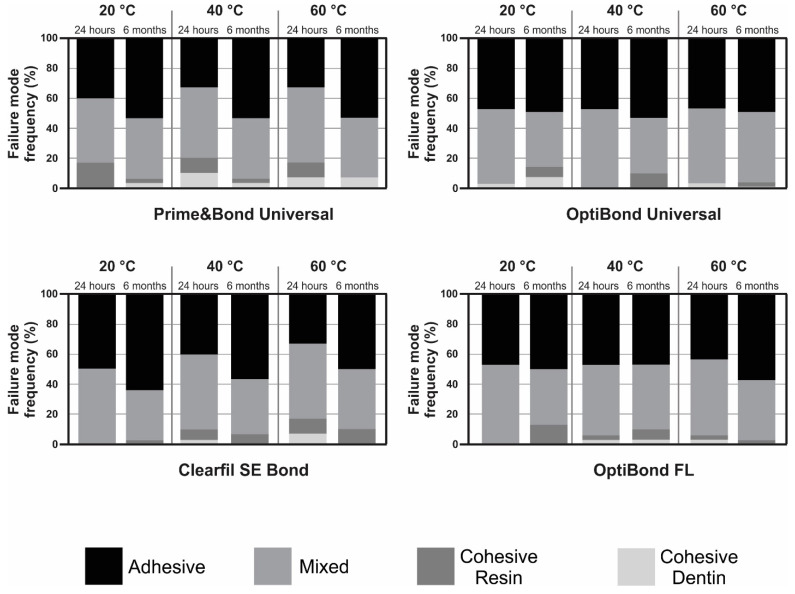
Failure mode distribution of the adhesives systems evaluated after bond strength test.

The results of the failure mode analysis for all the tested groups are presented in Figure 3. The evaluation of the failure modes indicated a predominantly adhesive or mixed failure across all temperature variations of air used for solvent evaporation with each adhesive system. A low prevalence of cohesive failure in resin or dentin was observed.

### 3.3. Scanning Electron Microscopy of Resin–Dentin-Bonded Interface

**Figure 4:**
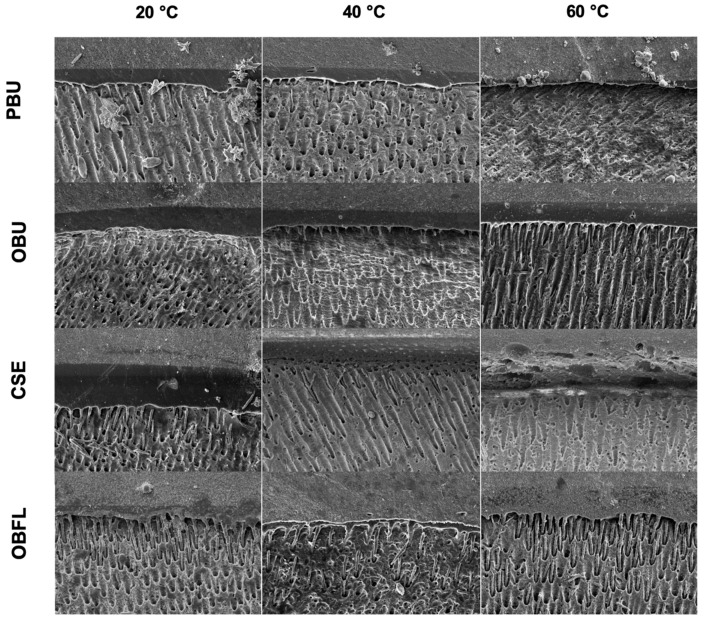
Scanning electron microscopy images of the restorative–dentin interface (×1500 magnification) show the tag formation and resin penetration for the four adhesive systems when using different air temperatures (20 °C, 40 °C, and 60 °C) for solvent evaporation. Prime&Bond Universal (PBU); OptiBond Universal (OBU); Clearfil SE Bond (CSE); and OptiBond FL (OBFL).

An ultrastructural observation of the restorative–dentin interface was performed for the four adhesive systems when using different air temperatures (20 °C, 40 °C, and 60 °C) for solvent evaporation (Figure 4).

The thickness of the adhesive layer and the creation of resin tags (RT) qualitatively varied amongst the temperatures evaluated. For OBFL, an elevated number of RT was observed between all the cold and warm air groups when compared to the other adhesive systems (PBU, OBU, and CSE). When passing from 20 °C to 60 °C, the thickness of the adhesive layer had a tendency to decrease, except for OBFL, where the adhesive layer was compact in all the temperatures employed. Moreover, the resin penetration increased for the three-step ER (OBFL), where remarkable infiltrations were seen at all the tested temperatures.

### 3.4. Solvent Evaporation Rate

**Figure 5:**
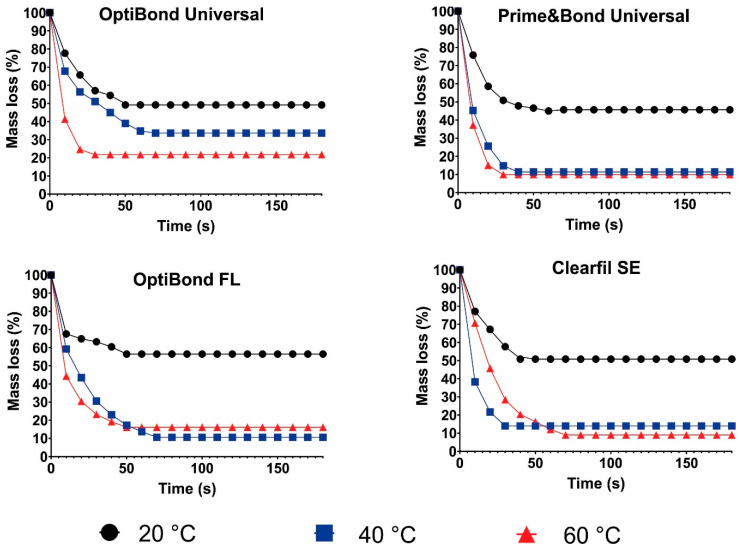
Solvent evaporation rate of the different adhesive systems tested according to the temperature of air stream.

The results of the solvent evaporation rate test are presented in Figure 5. For all adhesive systems tested, the use of 40 °C or 60 °C air for solvent evaporation led to an increased mass loss. At this temperature the mass loss for PBU, OBFL, and CSE reached around 90 wt%. On the other hand, when a temperature of 20 °C for air streaming was used, in all adhesives systems, the mass loss was around 50 wt%.

## 4. Discussion

For a successful dental restoration, it is essential to establish a robust chemical bond between the acidic functional monomer present in adhesives and the hydroxyapatite (HAp) within the dental substrate [1]. In light of the highly volatile nature of solvents, an adhesive can properly eliminate leftover water and regulate the solvent combined in an adhesive layer by means of air-blowing application for the attainment of a target adhesive performance [9]. Previous studies focused on air temperatures ranging from 37 °C to 80 °C as one of the most crucial factors of the air-blowing application [13,14,18]. Furthermore, as previously discussed, the gold-standard temperature of a warm air stream when applied to dentin should be between 50 °C and 60 °C [13]. Consequently, the 40 °C and the 60 °C were selected as the middle-level air temperatures in this present study. Therefore, this research aimed to study the influence of different air-drying temperatures (20 °C, 40 °C, and 60 °C) on the solvent evaporation of four adhesive systems to dentin. For this purpose, a prototype for a warm air stream was introduced. The results showed that the air temperatures affected the dentin BS of some of the adhesives used. Thus, this necessitates the rejection of the anticipated null hypothesis.

With all examined adhesives, at 24 h, only PBU displayed a significant variance in BS within the different temperatures used for solvent evaporation (*p* < 0.05). This difference was noticed between the 20 °C and 60 °C temperatures. Particularly, PBU is a 2-hydroxy ethyl methacrylate (HEMA)-free adhesive, and given the intricacy of its chemical composition, the in vitro presentation of UAs has been reported to be material-dependent [19]. In this current investigation, the absence of HEMA led to adverse effects on phase separation, primarily due to lingering water within the adhesive layer. Consequently, this condition resulted in a subpar bonding performance of PBU in the group exposed to a temperature of 20 °C. This was in line with a previous study where an HEMA-free adhesive showed a lower BS when compared to HEMA-based adhesives in 23 ± 2 °C groups [1]. In contrast, according to the previous research, solvent removal inside the adhesive layer of HEMA-free adhesives is considered to be an optimal option with higher temperatures up to 60 °C. Additionally, water (etching aid) and isopropanol (polarity adjustment) were considered in PBU as solvents [20]. It is likely that the isopropanol (a type of alcohol fully soluble in water) present in this adhesive (PBU) would ease adhesive spreading in between collagen fibers [21] at a higher temperature. Employing a warm air stream for solvent evaporation significantly improved the adhesive solutions’ ability to bind to dentin. The observed effect was likely due to the temperature increase within the substance, which facilitated solvent evaporation. The impact was especially obvious in adhesives based on alcohol/water [10]. This is in agreement with the discoveries of this present study. All in all, high temperatures (40 and to 60 °C) for the air delivery of solvent evaporation ameliorate the immediate BS performance of PBU to a dentin substrate.

For OBU, CSE, and OBFL, the rest of the comparisons were not statistically significant between all the temperatures tested after 24 h of aging (*p* > 0.05). Understanding that HEMA is used in the composition of almost all adhesives [4,5], it is essential to recognize the benefits and disadvantages of HEMA. This monomer, characterized by its low molecular weight and hydrophilic nature, demonstrates exceptional capability in penetrating and “wetting” dentinal substrates. Its hydrophilicity designates it as a significant adhesion-promoting agent, as it has been shown to enhance the initial bond strengths of adhesive systems. This enhancement occurs through an increased monomer penetration into dentin and the facilitation of a hybrid layer (HL) formation [4]. This is in harmony with the finding achieved in this study, as the OBU, CSE, and OBFL adhesives contain HEMA in their formulations. The co-solvents present in the formulation may also have an influence on the evaporation technique, since almost all HEMA-based adhesives were solvated with ethanol, which has a superior hydrogen bonding capacity (19.4 (J/cm^3^)1/2) than isopropanol (16.4 (J/cm^3^)1/2) within PBU [22]. So, undoubtedly, the adhesive layer of the HEMA-containing adhesives may have retained ethanol hydrogen (OBU and OBFL) and the hydrogen (CSE) bound to water, correspondingly to OBU, OBFL, and CSE employed in this research at any temperature, and reduced their degree of polymerization to dentin. Seemingly, the consistent bond strengths observed across the tested temperatures after 24 h could be attributed to the presence of water within the adhesive layer. This water may have been transferred to the surface and effectively removed by the air-blowing application, regardless of the temperature used (20 °C, 40 °C, and 60 °C). Considering this, it seems that the HEMA-contained adhesives were not sensitive to any temperatures employed in this research after 24 h.

Adhesives are often capable of producing HL with a strong instant and medium-term BS. Aging is frequently associated with the deterioration of dentin-bonded interface integrity and BS [23,24]. In this study, a 6-month storage in distilled water was performed. Following this aging, only CSE presented a significant difference between 20 °C and 60 °C air temperatures (*p* < 0.05). Starting with 40 °C, additional benefits were remarkable until achieving the highest BS at 60 °C. To clarify, CSE is a two-step SE adhesive system [25]. Indeed, water (CSE) and solvents deliver the ionization medium of the SE activity, and solvents lower their viscosity to facilitate resin infiltration into the porosities of the prepared tooth surface, as well as promote polymer chain mobility [16]. However, the remaining solvents inside the polymerized adhesive and HL might impede polymer network development [13,26]. This was not the case in the warm air groups (40 and 60 °C) tested for CSE after 6 months, so the clinician should emphasize the importance of an optimal strategy for enhancing the solvent evaporation and protecting the HL from any degradations. Solvents should be removed from adhesive systems as much as feasible prior to photopolymerization [16]. This is a key point because when the mixture of water and a solvent (water in the case of CSE) from the adhesive evaporates, the monomer density rises and becomes concentrated on the adhesive layer, reducing additional solvent vaporization [13]. However, when a warm air stream at roughly 60 °C was used, a considerable increase of around 20% in the resin–dentinal bond was noticed [12], and it was higher with the values of the present research. On a positive note, the use of warm air allowed for an increase in dentin’s BS of more than 50% than the cold air (an increase from 12.7 (5.0) to 26.7 (4.5) MPa with a warm air stream). The increase in the resin–dentin bonding is probably due to the improvement in the mechanical properties of the adhesive layer, which can be attributed to the higher rates of solvent evaporation [9]. This, perhaps, could be attributed to the fact that energy is released when heat is applied to the dentin. In the conditions of this study, the heat supplied by the warm air stream might have disrupted the molecular arrangement of the adhesive molecules. Correspondingly, the solvent evaporation rate increased from the bonding surface, permitting a higher resin–dentin bond to be achieved after 6 months of aging, as seen from the study results (for 40 and 60 °C).

CSE with a mild SE feature (pH of around 2) was used in this study for a partial demineralization of dentin [25], leaving a significant number of HAp crystals around the collagen fibrils [27]. Regarding the warm air groups, it is likely that 10-MDP and HAp may have interacted more chemically, and the BS increased accordingly. Further, it has been shown that removing HEMA from SE adhesives would reduce water sorption [28], but another research found that a 10% HEMA concentration would improve the adhesive system’s performance [29]. Considering that 20–40% of HEMA was present in the composition of the two-step SE “CSE” as described in their safety data sheet, the presence of extra %HEMA would have led to the formation of an aqueous and unstable gel that requires warm air application on the adhesive in order to achieve the optimal dentinal BS and stability with time. All things considered, CSE benefits from the warm air temperatures (definitely the 60 °C temperature) used in this study after 6 months of water storage.

Another notable discovery presumed from this analysis was that differences in BS between PBU, OBU, and OBFL for the different temperatures were not significant (*p* > 0.05) after 6 months of aging. While it is commonly observed that resin–dentin bonding reaches adequacy immediately, a decline in bonding effectiveness over time has also been documented [30]. However, these findings are not consistent with the results of this research, since despite the slight discrepancies in the mean values of BS, the maximum values were emphasized with warm air temperatures. Summarily, the adhesive BS remained consistent after 6 months of water storage with no changes. The chemical composition of the adhesive explains this plainly.

Because PBU is a HEMA-free adhesive, water contamination during the bonding process may initially lead to a decrease in dentin BS and the development of flaws within the adhesive layer and resin composite. This phenomenon occurs due to a phase separation between water and the hydrophobic components of the adhesive [20]. After 6 months of water storage, these flaws would result in less lasting bonding to dentin [31]. This was only applicable for the cold air group. As BS remained constant, this result confirms the study’s findings. For PBU, the results showed lower values when cold air was used, and these values remained lower after 6 months. For the warm air groups, especially the 60 °C temperature, higher BS values were shown after 6 months, associated with the same values at 24 h. This confirms the sensitivity of PBU to cold air and the resistance to the weakening of the BS with time when using warm air. Consequently, when employing the cold air temperature, it is advisable not to prioritize the use of the tested HEMA-free adhesive as the treatment of choice, and clinicians should opt for the warm air strategy with a 60 °C temperature. Moreover, the water content of the UAs varies [32]. The reduced water content may result in a hydrophobic adhesive surface with great resistance to hydrolytic breakdown. Previous research found that adhesives with more than 25% water suffered phase separation [33], and that low water contents in adhesive systems led to a superior pH and less chemical interaction with substrates [34]. That being so, adjusting the water content of adhesives prior to application to tooth surfaces might be critical. Even within the identical adhesive category, the ideas underlying the creation of UAs vary depending on the manufacturer. A water content of 10–15%, along with the presence of constituents considered to limit the impact of surface moisture, would be optimal for UAs [35]. As PBU contains 5–24.5% water [25], this could explain the non-significant discrepancy in BS between groups after 6 months.

For OBU, as acetone (highly volatile solvent) was present as 30–60% inside the bottle (with ~5–10% ethanol) [25], no significant difference in dentin BS was observed between the 6-month temperatures. Given that acetone has a relatively high vapor pressure, evaporation happens considerably faster in acetone-based adhesives than in ethanol-based adhesives, having less direct influence on BS [10]. Likewise, a prior study discovered that the solvent evaporation of experimental adhesives formulated with acetone remained similar when subjected to either room-temperature air stream or a 40 °C air stream [11]. These variables might explain why using a warm air stream for solvent volatilization did not increase the binding strength of acetone-based adhesives extensively [10].

There were no statistically significant differences in the BS values for the OBFL adhesive system (*p* > 0.05). This adhesive was based on glycero-phosphate dimethacrylate (GPDM) [25,36], which was not sensitive to any warm air stream used after 6 months, which rationalizes the results. Moreover, OBFL was the sole ER adhesive system evaluated in this investigation, demonstrating consistent bond strengths after 24 h and even after 6 months of water storage. This finding could be attributed to the unique characteristics of this product. OBFL has been previously recognized as the gold standard material due to its exceptional efficacy in both immediate and long-term BS tests [3], a finding corroborated by the current study. The presence of GPDM, which may engage in chemical interactions with HAp, and the densely filled bonding resin layer (48 wt%) applied over the primed dental surfaces, likely contribute to its superior performance, as indicated by this research [37].

Having this in mind, it appears that the ER strategy assured that this adhesive method remains a viable alternative with a correct air stream (60 °C air stream was the optimal choice when using this adhesive after the CSE). Another explanation of the values obtained for OBFL (ethanol and water-based adhesive) is that there is no direct link between the solvent content in the adhesive and the degree of cure [38]. This might hypothesize that OBFL contains a balanced mixture of solvents able to favor the warm air technique since a stable and high value was obtained with warmer temperatures.

Considering the effect of the adhesive system within each temperature (after 24 h and 6 months aging), there were no noteworthy variances among all the distinctive adhesive systems (*p* > 0.05). A possible explanation of the acquired results is that additional compositional variations, like initiators, percentage of water content, presence or absence of other functional monomers, such as 10-MDP, GPDM, among others, might be implicated in the bonding performance of such adhesives to dentinal substrates. These findings reveal that, while the solvent is an important determinant, other components in the adhesive formulation also influence the evaporation rate. The type of solvent and the mixing of components, counting HEMA (OBU, CSE, and OBFL), have been shown to impact the rate of solvent evaporation for commercial and experimental adhesives [39]. In spite of everything, regardless of the variation in components used in the adhesives tested in this study, the 60 °C temperature with almost the highest BS could be the reason for the formation of suitable adhesive layers.

To compare the BS values at 24 h and 6 months of aging, all of the air-drying temperatures (20 °C, 40 °C, and 60 °C) resulted in BS stability over time (*p* > 0.05). For the sub-optimal presentation of OBFL and CSE adhesive systems, it is theorized that BS results would be standardized following an extended period of aging. There is much documented success of these bonding agents, and it continues to be a gold standard in the category of SE and ER strategies [40,41,42]. Plus, the HL of UAs using the SE approach (like PBU and OBU used in this study) appears to last longer, as this bonding agent comprises functional monomers that can interact chemically with HAp and protect the collagen fibrils over time [24].

For PBU, all of the temperatures used resulted in BS stability. Regarding the cold temperature, the BS was directly lower and kept its low value with time. However, for warm temperatures, the dentinal BS was conserved, saving the higher dentinal long-term bond presentation. This unchanging value was due to the presence of dipentaerythritol pentaacrylate phosphate (PENTA) [43], 10-MDP [20,25], and methacrylamide monomers within the bonding arrangement [25,44]. In other investigations, adhesives including 10-MDP and PENTA demonstrated effectiveness in long-term clinical trials [45,46]. This was confirmed in this in vitro study. PENTA has been shown to be more stable than MDP in terms of BS retention at the end of the adhesive’s shelf life [43,47]. PBU maintained its stability in this regard. PBU is an HEMA-free adhesive and can help to efficiently eliminate water throughout the air-drying procedure [20]. Furthermore, methacrylamide monomers were synthesized as substitutes for HEMA in PBU to prevent phase separation and reduce water sorption within the adhesive [25,43,44,48]. The conclusion of this analysis is that PBU performed well at a high temperature of 60 °C immediately and after aging.

For OBU, the stability of the BS was observed in all the temperatures used with acceptable values in all the groups. Because of the presence of acetone in the composition of OBU, the BS could be reduced if not adequately evaporated [10,13,25,49]. It was shown in a previous investigation that an adhesive with a higher concentration of acetone (47% to 67%) reduced the values of resin–dentinal BS [50]. Knowing that OBU contains a similar % of acetone, the BS was stable. In addition, the presence of HEMA in OBU might make it vulnerable to degradation and water uptake [51]. However, this is not in agreement with this study as the BS remained stable; thus, solvent evaporation was made easy with the device presented in this research. Bearing that in mind, an adhesive containing a high concentration of acetone should be evaporated well and preferably with 40 °C warm air.

For CSE, all the 24 h values exhibited acceptable values. Yet, the stability of the BS was shown with time in all the temperatures employed, with a lower value for the cold air group after 6 months when compared to the higher value of 60 °C than 40 °C. The 10-MDP-based SE adhesive have better bond durability with aging [52]. This phenomenon was observed in the current study across all temperature variations. Plus, fillers present in the CSE increase BS and enhance the mechanical assets of an adhesive system [53]. Normally, appropriate resin–dentin bonding is usually directly reached as shown in the CSE groups, and minimized bonding effectiveness develops with time [30]. The drop in bonding was only seen in the 20 °C after 6 months and not significantly important to the 24 h value, but it was crucial when compared with the same adhesive with aging. This sheds light on the importance of aging on the BS of the adhesive to dentin. When selecting the CSE, a clinician can select the 60 °C warm air.

For OBFL, notwithstanding the small divergences in the mean values of BS, it can be concluded that solvent evaporation at elevated temperatures was higher, although not significantly so. According to a previous study [54], adhesive systems offering dental etchant, dental primer, and hydrophobic adhesive in each bottle without a solvent content in the adhesive are superior with regard to the adhesion stability, as long as the hydrophilic combinations have no influence on the degree of polymer conversion, with OBFL being an example of this [25,55]. This observation might help explain why the BS of OBFL did not decline between 24 h and 6 months across all the temperatures tested. As a requirement for an ER OBFL, a 60 °C warm air strategy will be the optimal decision.

Concerning the failures that happened following the μTBS testing, adhesive failure and mixed failures were the most common, while cohesive failures in the resin and dentin were less prevalent. This discovery is in agreement with a previous report by Taguchi et al. who found that warm air specimens had fewer blisters in the adhesive layer than the cold-air ones [12]. Precisely, the BS test employed a load force capable of passing through both the dentin and the resin composite prior to attaining the adhesive interfaces, resulting in an increased stress intensity at these areas [56]; thus, the number of mixed failures increased. It is worth mentioning that after the aging of 6 months, the failure analysis was frequently adhesive. This was due to the aging of the adhesive layer, which caused more adhesive fractures than the baseline mode of fracture (after 24 h) in the samples [25]. After aging, adhesive layer is prone to water uptake, which lowers the tensile strength and the stability of the material, and, consequently, hinders its ability to maintain the bonding of the dental structure to the restorative material [57]. By correlating each mode of adhesive failure with susceptibility to microleakage, risk of secondary caries, and restoration stability over time, clinicians can better understand the clinical implications of different bonding techniques and optimize treatment strategies to enhance the longevity and performance of adhesive restorations. In particular, the predominance of the adhesive or mixed failure modes observed in this study following BS tests between various adhesive systems and dentin underscores the intricate interplay between material properties and clinical application. While these modes of failure are indicative of immediate BS, their implications for long-term adhesive performance warrant a comprehensive examination.

Adhesive or mixed failures may raise concerns regarding the durability and longevity of adhesive restorations. In the context of warm air delivery, the prevalence of such failure modes suggests potential shortcomings in the adhesive interface’s resilience to environmental stressors. Prolonged exposure to oral conditions, including temperature fluctuations and mechanical stresses, could exacerbate these vulnerabilities, leading to compromised adhesive integrity over time. Addressing the underlying mechanisms driving adhesive failure modes is paramount for optimizing the longevity and clinical success of adhesive systems, thereby enhancing the durability of dental restorations and improving patient outcomes [58,59].

In this research, the resin–dentin interface of the three groups tested after 24 h was assessed through SEM. An elevated number of RT among all the cold and warm air groups was observed for OBFL when compared to the other adhesive systems (PBU, OBU, and CSE). Therewith, resin penetration increased for this three-step ER (OBFL), where distinguished infiltration was seen in all the temperature tested. The difference in infiltration and tag formation obtained for OBFL was, perhaps, linked to the unique feature of the etching step that preceded the resin infiltration [25]. This etching phase is important in facilitating deeper adhesive penetration into the dentinal substrate, leading to prolonged RT and a thicker HL development [54]. Taking this into account, regardless of the temperature used for solvent evaporation, using phosphoric acid for eliminating the smear layer and smear plugs resulted in an increased adhesive penetration. On the other hand, when the temperature of air increased from 20 °C to 60 °C, the thickness of the adhesive layers seemed to have a tendency to decrease, except for OBFL, where the adhesive layer was compact in all the temperatures employed. The formation of dense adhesive layers was accomplished due to the ability of warm air blowing to effectively evaporate the solvent contents of the adhesive systems [60]. However, the BS of the resin–dentin interface has been observed to be independent of the thickness of the HL and/or adhesive layer [61]. The current results imply that the bonding performance, particularly degradation during extended water storage, may vary depending on the chemical compositions of adhesives and the type and quality of the functional monomer present. This influence persists regardless of factors such as the adhesive layer thickness and the extent of tag penetration into dentinal tubules. The dentinal tubule orientation varies between samples, as seen from micrographs. It should be highlighted that tubule diameters and density increase from the dentino–enamel junction to the middle dentin region [62].

Gas chromatography serves as a reliable method for determining the presence of solvents in dental adhesive systems [63]. Through a precise analysis, it enables researchers to assess the impact of warm air utilization on solvent evaporation kinetics, contributing to a deeper understanding of adhesive performance and formulation optimization. In this study, the solvent evaporation rate was used for recording the mass loss of solvents in adhesive systems. So, another explanation of the results from the solvent evaporation rate outcomes would be the type of solvents present in each primer or adhesive tested. For all adhesive systems examined, the use of 40 °C or 60 °C air for solvent evaporation led to an augmented mass loss. At these temperatures, the mass loss for PBU, OBFL, and CSE reached around 90 wt%. Various molecules exhibit differing degrees of attraction between them. By way of explanation, the common attraction among water molecules (in PBU, CSE, and OBFL) and alcohol molecules (in PBU, OBU, and OBFL) is higher compared to acetone (OBU), as it engages hydrogen bonding forces. That being the case, ethanol (with a vapor pressure that is halfway between that of acetone and water) and water have higher boiling temperatures and vapor pressures than acetone, making evaporation more difficult [9,10,13]. This explains why warmer temperatures facilitated the evaporation of water/alcohol and water by increasing their mass loss. Thus, the application of heat via a warm air stream could facilitate the removal of water from the composition more efficiently. On the other hand, from the data on mass loss during solvent evaporation, when a temperature of 20 °C for air streaming was used, in all adhesives systems, the mass loss was around 50 wt%. The warm air stream removed an expressively higher quantity of solvents from all the tested adhesives when compared to normal air-blowing in a previous investigation [12]. This discovery reinforces the conclusion of this study that warm air drying expedited and simplified solvent vaporization, resulting in advantages for enhancing dentinal BS.

This study is subject to certain limitations that warrant consideration when interpreting the results. First, the inclusion of various quantities and types of adhesive systems introduced potential confounding factors, making it challenging to isolate the specific effects of individual adhesives. Furthermore, the lack of detailed information regarding the exact composition of the adhesive systems used added complexity to the results’ interpretation, hindering the ability to attribute observed outcomes to specific components. This uncertainty limited the depth of the analysis and the precision with which conclusions could be drawn. Moreover, in forthcoming studies, it may be beneficial to explore storage options such as water, artificial saliva, or alternative accelerated aging solutions prior to evaluating BS [17]. Including multiple storage times in the experimental design can enhance its rigor and coherence, and this approach could be considered for future analyses. The µTBS method for dentin testing presents challenges including a time-consuming sample preparation and potential damage to the dentin structure, while the interpretation of results requires expertise and a consideration of various factors. Nevertheless, Beloica et al. [64] noted that the adhesive system significantly influences BS irrespective of the testing methodology employed. Adhesion to tooth structure should ideally give retentive strength, provide a marginal seal, be simple to create, and be clinically durable. The authors recommend the µTBS test, particularly following a durability challenge, as a suitable surrogate assessment of dental composite restorative retention (direct restoration). More sophisticated techniques should be employed to assess solvent evaporation, such as gas chromatography [63]. Incorporating quantitative measurements of solvent evaporation rates and their correlation with bonding efficacy would strengthen the validity of the findings. Subsequent studies could employ a precise, safe, and non-destructive technique such as scanning confocal microscopy for the evaluation of the resin–dentin interface [65]. Further studies could explore the temperature rise in the pulp chamber when employing warm air to better understand the practical implications and potential limitations associated with using higher air temperatures in dental procedures. Additionally, in a clinical context, the difficulty in standardizing the temperature of the air emitted from the triple syringe presents a practical challenge. This variability in environmental conditions may compromise the accuracy of the experimental controls, reflecting a potential mismatch between idealized laboratory settings and the dynamic nature of real-world clinical scenarios. Validation procedures, including testing methodologies and results, would be essential for assessing this device’s efficacy and reliability in delivering warm air for adhesive applications.

## 5. Conclusions

In summary, higher air-blowing temperatures (40 °C and 60 °C) during application could potentially enhance the dentinal BS of adhesive systems (PBU, OBU, CSE, and OBFL), a variable influenced by specific materials used in clinical settings, indicating the crucial role of warmer temperatures in improving the quality of the HL and promoting better interaction between adhesives and dentin tissue.

## Figures and Tables

**Table 1:** Manufacturers and compositions of the adhesives tested in this current research.

Adhesive and Manufacturer	Classification of the Material	Main Composition *	Adhesive Application Protocol
PBU (Dentsply DeTrey GmbH, Konstanz, Germany)	Mild Universal pH = 2.5	10-MDP, PENTA, isopropanol, water, photoinitiator, bi- and multifunctional acrylate	Using a microbrush applicator, dispense one drop of PBU to all dentinal surfaces. Avoid pooling. Keep PBU slightly agitated for 20 s. Evaporate solvent with air delivered from the device (cold or warm) for at least 10 s. Light-irradiate for 20 s.
OBU (Kerr Co, Orange, CA, USA)	Universal pH = 2.5–3.0	Acetone, HEMA, GDMA, ethanol, GPDM	Using a microbrush applicator, a generous amount of OBU adhesive is applied to the dentinal surfaces. Scrub the surface with a brushing motion for 20 s. Dry the adhesive with air delivered from the device (cold or warm) for at least 10 s. Light-irradiate for 20 s.
OBFL (Kerr Co, Orange, CA, USA)	Three-step etch-and-rinse pH primer: 1.9; pH bonding: 6.9	Etchant: 37.5% H_3_PO_4_ Primer: HEMA, GPDM, MMEP, water, ethanol, CQ, and BHT Adhesive: Bis-GMA, HEMA, GDMA, CQ, and filler (fumed SiO_2_, barium aluminoborosilicat, Na_2_SiF_6_), coupling factor A174	Using a microbrush applicator, apply OBFL primer over dentin surfaces with a light scrubbing motion for 20 s. Dry the primer with air delivered from the device (cold or warm) for at least 10 s. At this point, the dentin surface should have a slightly shiny appearance. Using a new microbrush applicator, apply OBFL adhesive to the prepared dentin surfaces with a light scrubbing motion for 20 s, creating a thin coating. Gently air-dry for approximately 5 s from the device (cold air). Light-irradiate for 20 s.
CSE (Kuraray Noritake Dental Inc., Tokyo, Japan)	Two-step self-etch pH primer = 1.76 pH bond = 2	Primer: 10-MDP, HEMA, hydrophilic dimethacrylate, CQ, DEPT, water Bond: MDP, HEMA, Bis-GMA, hydrophobic dimethacrylate, CQ, DEPT, silanized colloidal silica	Using a microbrush applicator, apply primer for 20 s. Dry with air delivered from the device (cold or warm) for at least 10 s. Using a new microbrush applicator, apply bond. Apply air flow gently from the device (cold air). Light-irradiate for 20 s.

* Based on companies’ MSDS. 10-MDP = 10-methacryloyloxydecyl dihydrogen phosphate; PENTA = dipentaerythritol pentaacrylate phosphate; HEMA = 2-hydroxy ethyl methacrylate; GDMA = glycerol-dimethacrylate; GPDM = glycero-phosphate dimethacrylate; MMEP = methacryloyloxy-ethyl-dihydrogen phosphate; CQ = camphorquinone; BHT = butyl hydroxy toluene; Bis-GMA = bisphenol A-glycidyl methacrylate; DEPT = N,N-diethyl-p-toluidine; Clearfil SE Bond (CSE); OptiBond FL (OBFL); OptiBond Universal (OBU); Prime&Bond Universal (PBU).

**Table 2:** Mean and standard deviation of the micro-tensile bond strength test (MPa) of the different temperatures used for solvent evaporation for each adhesive system test after 24 h of aging.

Temperature for Solvent Evaporation	Prime&Bond Universal	OptiBond Universal	OptiBond FL	Clearfil SE
20 °C	^A^ 13.3 (3.4) ^a^	^A^ 20.9 (3.1) ^a^	^A^ 20.5 (5.5) ^a^	^A^ 20.5 (5.5) ^a^
40 °C	^A^ 22.1 (9.9) ^ab^	^A^ 26.8 (7.6) ^a^	^A^ 29.3 (4.5) ^a^	^A^ 26.2 (8.2) ^a^
60 °C	^A^ 24.6 (4.1) ^b^	^A^ 24.2 (3.8) ^a^	^A^ 29.9 (6.3) ^a^	^A^ 28.6 (10.4) ^a^

Different lowercase letters indicate the presence of statistically significant differences between temperatures for solvent evaporation within each adhesive system (*p* < 0.05). Different uppercase letters indicate the presence of statistically significant differences between adhesive systems within each temperature for solvent evaporation (*p* < 0.05).

**Table 3:** Mean and standard deviation of the micro-tensile bond strength test (MPa) of the different temperatures used for solvent evaporation for each adhesive system test after 6 months of aging.

Temperature for Solvent Evaporation	Prime&Bond Universal	OptiBond Universal	OptiBond FL	Clearfil SE
20 °C	^A^ 12.69 (6.6) ^a^	^A^ 16.08 (7.3) ^a^	^A^ 16.0 (7.4) ^a^	^A^ 12.7 (5.0) ^a^
40 °C	^A^ 19.24 (6.1) ^a^	^A^ 24.3 (5.99) ^a^	^A^ 22.1 (11.4) ^a^	^A^ 22.5 (6.1) ^ab^
60 °C	^A^ 19.61 (8.6) ^a^	^A^ 21.47 (6.6) ^a^	^A^ 26.2 (6.6) ^a^	^A^ 26.7 (4.5) ^b^

Different lowercase letters indicate the presence of statistically significant differences between temperatures for solvent evaporation within each adhesive system (*p* < 0.05). Different uppercase letters indicate the presence of statistically significant differences between adhesive systems within each temperature for solvent evaporation (*p* < 0.05).

**Table 4:** Mean and standard deviation of the micro-tensile bond strength test (MPa) of the different temperatures of air used for solvent evaporation for the adhesive systems tested at 24 h and 6 months of aging.

Adhesive/Temperature	Aging	
Prime&Bond Universal	24 h	6 months
20 °C	13.3 (3.4)	12.69 (6.6)
40 °C	22.1 (9.9)	19.24 (6.1)
60 °C	24.6 (4.1)	19.61 (8.6)
OptiBond Universal	24 h	6 months
20 °C	20.9 (3.1)	16.08 (7.3)
40 °C	26.8 (7.6)	24.3 (5.99)
60 °C	24.2 (3.8)	21.47 (6.6)
Clearfil SE	24 h	6 months
20 °C	20.5 (5.5)	12.7 (5.0)
40 °C	26.2 (8.2)	22.5 (6.1)
60 °C	28.6 (10.4)	26.7 (4.5)
OptiBond FL	24 h	6 months
20 °C	20.5 (5.5)	16.0 (7.4)
40 °C	29.3 (4.5)	22.1 (11.4)
60 °C	29.9 (6.3)	26.2 (6.6)

There were no statistically significant differences in any comparison between 24 h and 6 months (*p* > 0.05).

## Data Availability

The data presented in this study are available upon reasonable request from the author (R.B.).
